# Niosome as a Drug Delivery Carrier for Sorafenib: Preparation, Investigation of Physicochemical Properties, and *In Vitro* Effects on HepG2 Cell Line

**DOI:** 10.34172/apb.43228

**Published:** 2024-10-02

**Authors:** Mohammad Amin Raeisi Estabragh, Behzad Behnam, Masoud Torkzadeh-Mahani, Abbas Pardakhty

**Affiliations:** ^1^Student Research Committee, Kerman University of Medical Sciences, Kerman, Iran.; ^2^Pharmaceutics Research Center, Institute of Neuropharmacology, Kerman University of Medical Sciences, Kerman, Iran.; ^3^Herbal and Traditional Medicines Research Center, Kerman University of Medical Sciences, Kerman, Iran.; ^4^Department of Pharmaceutical Biotechnology, Faculty of Pharmacy, Kerman University of Medical Sciences, Kerman, Iran.; ^5^Department of Biotechnology, Institute of Science, High Technology and Environmental Sciences, Graduate University of Advanced Technology, Kerman, Iran.; ^6^Department of Pharmaceutics, Faculty of Pharmacy, Kerman University of Medical Sciences, Kerman, Iran.

**Keywords:** HepG2 cell line, Niosome, Sorafenib, Target drug delivery

## Abstract

**Purpose:**

Sorafenib is known as one of the oral anti-cancer drugs used in liver cancer. However, its lipophilic nature can lead to side effects, variable pharmacokinetics, and poor absorption. The use of novel drug delivery systems, such as niosomes, may help address these issues and improve the effectiveness of sorafenib.

**Methods:**

Different niosomal formulations of sorafenib were prepared. The morphology, size analysis, and physical stability were investigated. The encapsulation efficiency percent of the selected formulations was measured using the dialysis method, and the release of sorafenib was checked for four hours using the Franz diffusion cell. The cytotoxicity and *in vitro* effect on the HepG2 cell line was investigated using the MTT assay and flow cytometry.

**Results:**

The mean volume diameter of Span 60/Tween 60/cholesterol (45/45/10 mole%) niosomal formulation was 6 µm with minimal size changes and good stability over six months of storage. The encapsulation efficiency percent of this formulation was 66.40±1.11, and 61.43±1.42 percent of the drug was released within 4 hours. *In vitro* release followed Higuchi kinetics. Cytotoxicity tests showed an IC_50_ of 7.5 µg/mL for the niosomal formulation, compared to 15.96 µg/mL for the sorafenib solution.

**Conclusion:**

Niosomes containing Span 60/ Tween 60/ cholesterol (45/45/10 mole%) are promising for loading and sustained release of sorafenib. The use of niosome as a carrier can enhance the effectiveness of sorafenib on the HepG2 cell line. This niosomal formulation of sorafenib shows potential for future studies.

## Introduction

 According to estimates from the World Health Organization (WHO), cancer is one of the common causes of death.^1^ Cancer will cause approximately 20 million new cases and 9.7 million deaths in 2022. In addition to those who survive cancer for at least five years, there are estimated to be 53.5 million of those living with the disease.^2^ More than 8% of deaths are caused by various cancers, and the second rank of cancer in men is related to liver cancer.^1^ There are different methods to treat cancers.^3^ Chemotherapy, as one of the most efficient methods for cancer treatment, is one of the first options for different types of cancer.^4^ In particular, angiogenesis plays a crucial role in the spread and establishment of metastatic tumor cells, making combination therapies, such as angiogenesis-inhibitory treatments, essential for preventing the spread of cancer.^5^

 Sorafenib (SB) is an oral multikinase and vascular endothelial growth factor receptor inhibitor that suppresses cancer cell proliferation, and angiogenesis. This mechanism leads to increased cancer cell apoptosis. SB reduces tumor growth by inhibiting the activity of Raf-1, B-Raf and signaling in the Ras/Raf/MEK/ERK pathway, also by targeting hepatocyte factor receptor (c-Kit), Fms-like tyrosine kinase (FLT-3), vascular endothelial growth factor receptor (VEGFR)-2, VEGFR-3, and platelet-derived growth factor receptor (PDGFR-β) inhibit tumor angiogenesis.^6,7^ This drug was approved by the FDA in 2006 as a treatment for advanced renal cell carcinoma, and in 2007 for treating advanced hepatocellular carcinoma.^6,7^ However, fluctuations in its pharmacokinetics due to its low solubility and side effects such as diarrhea, increased blood pressure, fatigue, anorexia, coronary artery spasm, and gastrointestinal bleeding have affected its clinical use.^8^ Various lipid-based formulations of SB such as liposome and pH-sensitive liposome have been prepared. Their positive effect in reducing side effects and increasing the therapeutic efficacy of SB has been observed.^9-11^

 Encapsulation of the drugs in lipid-based carriers as drug delivery systems offers several advantages. These include increased drug solubility, improved the pharmacokinetics and pharmacodynamics properties, and the controlled and continuous release of the drug. Additionally, the drug is protected from the reticuloendothelial system, allowing for a longer presence in the blood circulation and increasing the likelihood of reaching the desired location, such as cancer tissue and cells.^4^ Various types of lipid-based carriers, such as liposomes, niosomes, and solid lipid nanoparticles, have shown positive effects in delivering anti-cancer drugs, including angiogenesis-inhibiting drugs for cancer treatment.^4,12-14^

**Figure 1:**
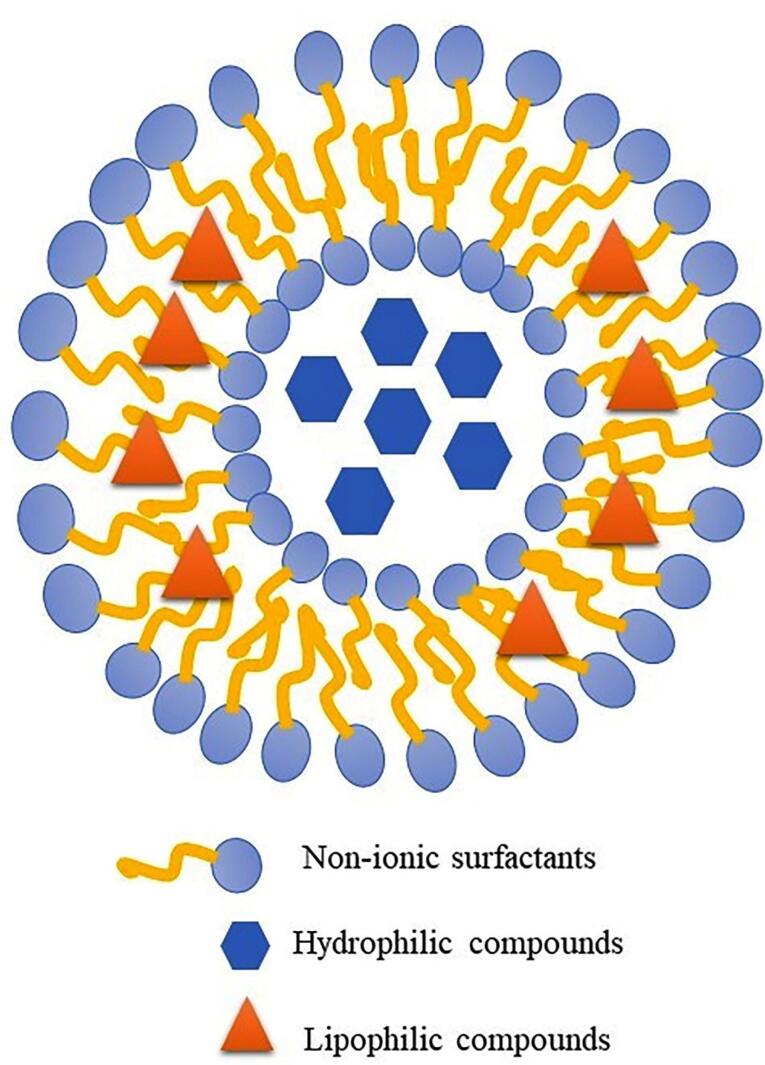
Schematic structure of niosome as a bilayer lipid carrier for drug delivery of lyophilic and hydrophilic therapeutic agent

 Niosomes are lipid vesicle carriers that are prepared using non-ionic surfactants such as sorbitan esters (Span^®^) and their polyxylated derivatives (Tween^®^), and additives such as cholesterol, which are used to increase lipid bilayers stability (Figure 1).^15,16^ Compared to liposomes, niosomes have advantages such as more stable and cheaper non-ionic surfactants compared to phospholipids as the main components of liposomes.^15,17^ There are different methods for preparing niosomes: thin-film hydration, ether injection, reverse phase evaporation, solvent evaporation from the double emulsion, microfluidic, etc. have been used to prepare niosomes.^15^ Thin-layer film hydration is one of the most common methods for niosome preparation.^17^

 In this study, niosomes containing SB, an anticancer drug, were prepared using the thin film hydration method. The aim was to create a novel drug delivery system that would not only control the release of the drug, but also address the issues of low solubility and potential side effects of SB, ultimately increasing its effectiveness.

## Materials and Methods

###  Materials

 Materials used in this research are SB as a gift (The Parsian Pharmaceutical Company, Tehran, Iran), non-ionic surfactants as Span^®^ 20, 40, and 60 and Tween^®^ 20, 40, and 60 (Fluka Company, Buchs, Switzerland), Cholesterol (Sigma-Aldrich, St. Louis, USA), Fetal bovine serum (FBS) and low-glucose Dulbecco’s Modified Eagle’s Medium (DMEM) (Biosera, Cholet, France), Chloroform, Ethanol, and Isopropyl alcohol as organic solvent (Merck Chemical Company, Darmstadt, Germany). The HepG2 cell line (The Pasteur Institute, Tehran, Iran) was also used in this study.

###  Methods

####  Preparation of SB niosomes

 The film hydration technique was used to prepare niosomal suspensions.^18^ To prepare SB niosomes, Spans^®^ (20, 40, and 60) and Tweens^®^ (20, 40, and 60) mixture were used in equimolar proportions, along with varying molar ratios of cholesterol as outlined in Table 1. The final concentration of SB was 100 µg /mL, and it was dissolved in a mixture of chloroform and methanol (90:10 v/v) along with the surfactants and cholesterol. The solvent was evaporated using a rotary evaporator device (Heidolph, Germany) at a temperature of 55 °C. The dried lipid film was mixed with normal saline (NS) and rotated at 180 rpm and 55 °C for 30 minutes. The resulting lipid vesicles were stored in borosilicate glass (type I) vials at room temperature for 24 hours before being transferred to a refrigerator for further analysis.

**Table 1:** Composition of different niosomal formulations containing SB

**Name**	**Constituents of the lipid phase**	**Molar %**
F 1	Span20/Tween20cholesterol	25/25/50
F 2	Span20/Tween20/cholesterol	35/35/30
F 3	Span20/Tween20/cholesterol	45/45/10
F 4	Span40/Tween40/cholesterol	25/25/50
F 5	Span40/Tween40/cholesterol	35/35/30
F 6	Span40/Tween40/cholesterol	45/45/10
F 7	Span60/Tween60/cholesterol	25/25/50
F 8	Span60/Tween60/cholesterol	35/35/30
F 9	Span60/Tween60/cholesterol	45/45/10

####  Morphological investigation, size analysis, and physical stability

 Niosomes, such as the other lipid vesicular systems, could further be classified into multilamellar vesicles (MLV), unilamellar vesicles (LUV), and small unilamellar vesicles (SUV). The morphology, aggregation, and separation of vesicle constituents were characterized using a light microscope (Leitz HM-LUX3, Germany). The microscope was equipped with a digital camera, and the images were captured at a magnification of × 400. The size of vesicles (the mean volume diameters; d_V50_) was analyzed using the static laser light scattering technique (Malvern MasterSizer 2000E, UK). For evaluation of the physical stability, the formulations were stored at 4-8°C. The size analysis was conducted at specific time intervals: one week, one month, 3, and 6 months after storage of niosomes at refrigerator temperature. The span that represents dispersity (from almost monodisperse to highly polydisperse) was calculated by the following equation^17^:


Eq. 1 $$Span=\frac{d_{v90}-d_{v10}}{d_{v50}}$$

In which d_V90_, d_V50, _and d_V10_ are cumulative 90, 50, and 10 percent undersize volume size distributions, respectively.

####  Determination of SB concentration

 UV spectrophotometry was used to determine the amount of SB.^19^ The standard solution of SB (10 µg /mL) in a mixture of ethanol and water was prepared, and it was scanned at the wavelength of 200 to 400 nm by UV spectrophotometer (UV/Visible Spectrophotometer Optizen 3220, South Korea). After determining the maximum absorption wavelength (λ_max_), standard solutions of SB (2-10 µg /mL) were prepared, and their absorption was measured at the λ_max_. The graph of absorption against concentration was drawn using Microsoft Office Excel^®^ software, and its equation was obtained. Also, accuracy, precision, limit of determination (LOD), and limit of quantification (LOQ) were checked, and calculated.^20^ The following equations are used for LOD and LOQ calculation:


Eq. 2 $$S_{y/x}=\sqrt{\frac{\sum_{i}{\left(y_{i}-{\hat{y}}_{i}\right)}^{2}}{n-2}}$$

Eq. 3 $$LOD=\frac{3\;S_{y/x}}{Slope}$$

Eq. 4 $$LOQ=\frac{10\;S_{y/x}}{Slope}$$

*S*_*y/x*_ is the standard deviation of the data, y is the measured response, ${\hat{y}}_{i}$ is the response obtained by the line equation, and n is the number of points.

####  Encapsulation efficiency percent (EE%) measurement 

 The dialysis method was used to separate free SB from the encapsulated drug.^21^ In this method, one mL of the niosomal formulation was placed in a cellulose acetate dialysis bag (Visking tube, MW cut off 12 KD) and exposed to a mixture of ethanol and water (80:20 v/v) for four hours at room temperature. The concentration of the free drug that permeated through the membrane was determined using UV spectroscopy. By adding one ml of isopropyl alcohol to the niosomal suspension and disrupting the niosomal bilayers, the amount of SB encapsulated in the niosomes was also determined. The EE% was calculated using the following equation:


Eq. 5 $$EE\%=\frac{C_{E}}{C_{E}+C_{F}}\times 100$$

C_E_ and C_F_ refer to the quantity of SB contained within niosomes and not encapsulated (free), respectively.

####  In vitro release study

 The release rate of SB niosomes was evaluated using a Franz diffusion cell (15 mL) at 37 °C.^22^ A cellulose acetate dialysis tube with a molecular weight cutoff of 12 KD was utilized as the artificial membrane. Prior to use, the membrane was hydrated in a mixture of ethanol and water (80:20 v/v) in the recipient phase overnight. Samples were collected at various time points and after each sampling, the receptor phase was replenished with fresh solvent. The cumulative percentage of SB released was plotted against time. To determine the most appropriate model for the release of SB niosomes, various kinetic models including zero order, first order, Higuchi, Peppas, and Hixon-Crowell were assessed.^17,23^

####  Cell culture, cytotoxicity assay, and flow cytometry analysis

 The cells were grown in a DMEM medium containing 10% heat-inactivated FBS and 1% penicillin/streptomycin. They were incubated at 37°C in a humidified incubator with 5% CO_2_. All experiments were performed on the HepG2 cell line with passage numbers ranging from 1 to 10. In a 96-well cell culture plate, HepG2 cells (10 000 cells per well) were seeded and allowed to attach overnight at 37 ºC in an incubator.^24^ After attachment, the culture medium was replaced with 100 µL of fresh growth medium containing different concentrations (0.625-40 µg /mL) of SB solution and SB niosomes (5 wells per dose). The cells were then allowed to grow for 24 hours at 37 °C and in a humidified 5% CO_2_ environment. Cell viability after adding niosomal formulations and solutions was assessed using the MTT assay.^25^ Briefly, 20 µL of MTT solution (5 mg /mL in PBS) was added to each well, and the plate was incubated at 37ºC for an extra three hours. After removing the culture medium, 100 µL of DMSO was added to each well to dissolve the formazan crystals. Finally, the absorbance was measured at 570 nm against a blank (DMSO and empty niosome). The percentage of cell viability was defined by calculating the ratio of the absorbance of the sample to the control. The median inhibition concentrations (IC_50_) of the SB solution and SB niosomes were then determined by GraphPad Prism^®^ software.^24,26^

 To prepare cells for flow cytometry studies, different treatments (IC_50_ of SB solution and SB niosomes) were used to incubate with HepG2 cells. After incubation (24 hours at 37 °C and in a humidified 5% CO_2_ environment), the cells were washed with PBS and trypsinized. They were then collected in a fresh medium containing 10% FBS and centrifuged at 200 × g for 5 min at 4 ºC. The supernatant was discarded, and the cell pellets were rewashed with PBS. Afterward, they were centrifuged for 5 minutes at 500 × g at 4 ºC for flow cytometry analysis. The flow cytometry analysis used the Annexin V/ Propidium Iodide (PI) apoptosis detection kit (Miltenyi Biotec, Germany). In this analysis, the cell suspensions were incubated with FITC-conjugated annexin V and propidium iodide in the dark for 15 minutes on ice. Then, the ice-cold binding buffer was added. Within 30 minutes, the cell preparations were analyzed using a flow cytometer (BD-Biosciences, USA) and FlowJo v7.6.1 software (FlowJo, LLC).^24,27^

###  Statistical analysis

 Statistical analysis was conducted using GraphPad Prism software (Prism for Windows, Version 9, GraphPad, Dotmatics). To compare groups, a one-way ANOVA followed by Tukey’s Multiple Comparison test was performed with a significance level set at 0.5 (*P* < 0.05).

## Result and Discussion

###  Morphology, size distribution, and physical stability

**Figure 2:**
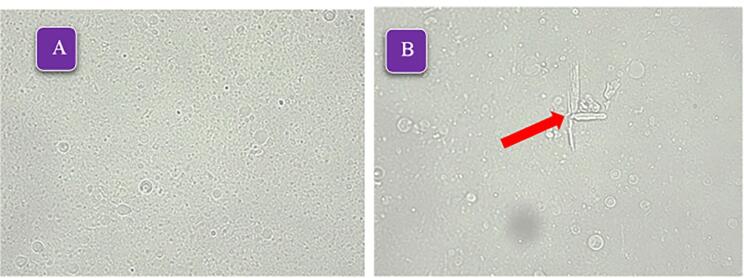
The light microscope micrographs ( × 400) of formulations A: F9 (Span60/Tween60/cholesterol 45/45/10 mole%), B: F4 (Span20/Tween20/cholesterol 25/25/50 mole%), the arrow indicates the presence of cholesterol crystals and lack of proper stability

 All prepared formulations successfully formed niosomes, as shown in Table 1. Notably, formulations containing Span 60/Tween 60 had a higher number of formed niosomes compared to the other formulations. This can be attributed to the lower HLB value of Span 60/Tween 60 (9.8) compared to Span 40/Tween 40 (11.15) and Span 20/Tween 20 (12.65) formulations. Additionally, the shorter chain length of the surfactants in Span 60/Tween 60 resulted in smaller niosomes. This phenomenon has been observed in previous studies as well.^28,29^ The most formed niosomes are MLV type, consistent with previous studies according to the preparation method of thin layer hydration.^30,31^ With the increase in the percentage of cholesterol in different formulations, especially the formulations containing Span 40/Tween 40, some crystals were observed, which is probably due to the competition of the SB as a lipophilic drug and cholesterol to be placed in the space of the lipid bilayer.^32,33^ Due to the appropriate number of formed niosomes and the absence of crystals, the formulations containing Span 60/Tween 60 were selected for further study. Figure 2 shows the light microscope images ( × 400) of formulations F4 and F9.

**Figure 3:**
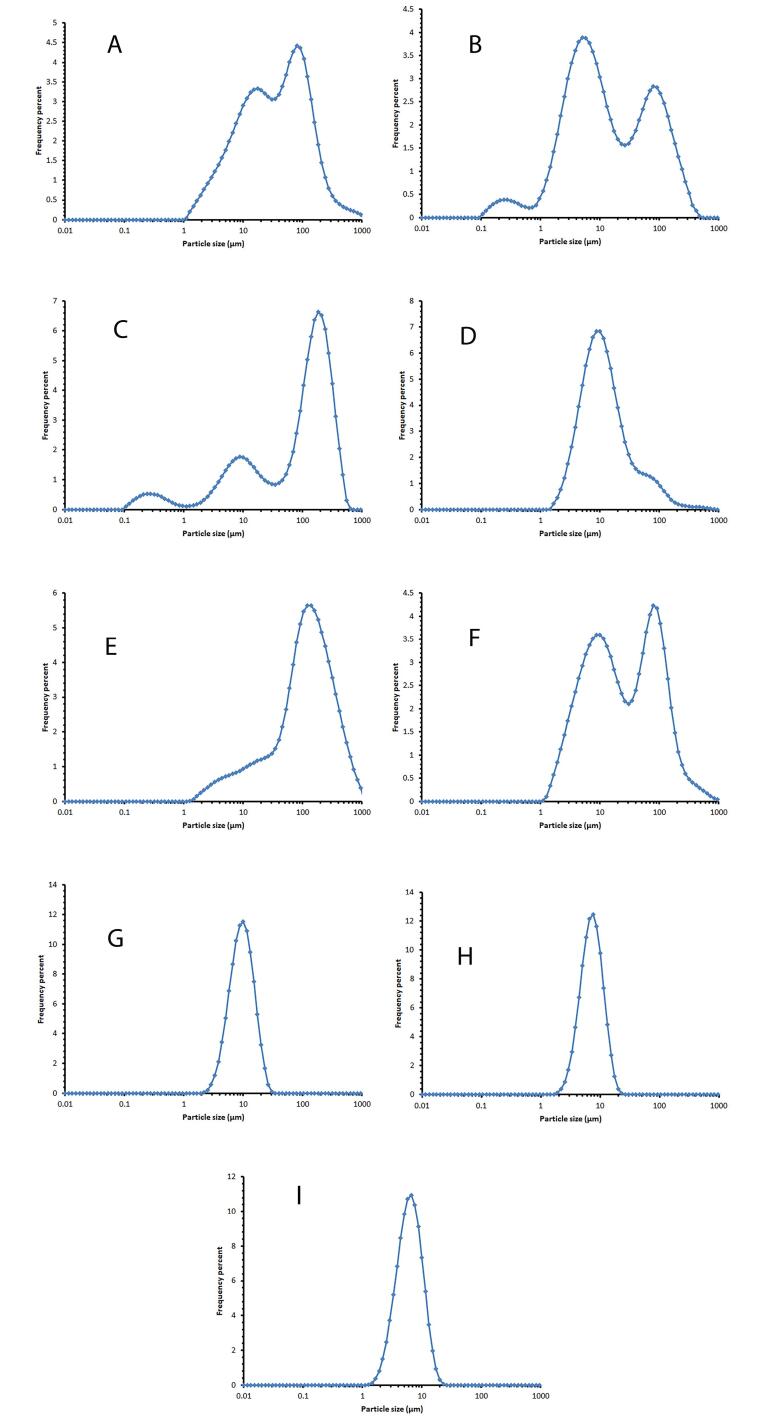
The vesicle size distribution diagram of the selected formulation one week after preparation and storage at 2-8°C. A: F1 (Span20/Tween20/cholesterol 25/25/50 mole%), B: F2 (Span20/Tween20/cholesterol 35/35/30 mole%), C: F3 (Span20/Tween20/cholesterol 45/45/10 mole%), D: F4 (Span40/Tween40/cholesterol 25/25/50 mole%), E: F5 (Span40/Tween40/cholesterol 35/35/30 mole%). F: F6 (Span40/Tween40/cholesterol 45/45/10 mole%), G: F7 (Span60/Tween60/cholesterol 25/25/50 mole%), H: F8 (Span60/Tween60/cholesterol 35/35/30 mole%), I: F9 (Span60/Tween60/cholesterol 45/45/10 mole%)

**Figure 4:**
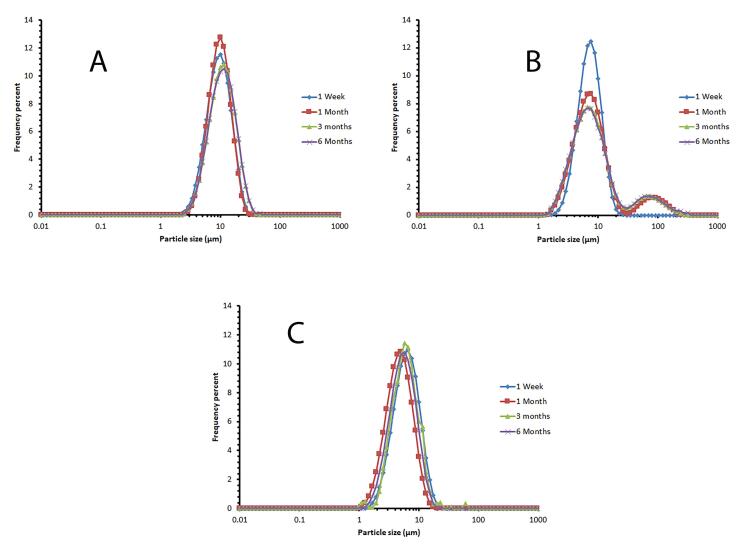
The vesicle size distribution diagram of the selected formulation during 6 months of storage at 2-8 °C, as an indicator for niosomal physical stability. A: F7 (Span60/Tween60/cholesterol 25/25/50 mole%), B: F8 (Span60/Tween60/cholesterol 35/35/30 mole%), C: F9 (Span60/Tween60/cholesterol 45/45/10 mole%)

 The vesicle size distribution of the selected formulations showed a log-normal and bell-shaped distribution in the first week. Over time, an increase in size was observed in formulation F8, which could be due to small niosomes incorporated together. Adding cholesterol to lipid bilayers enhances their stability and changes the phase transition from solid to liquid-ordered. Cholesterol modifies the flexibility of chains within bilayers, leading to a wider lipid bilayer and consequently an increase in vesicle size.^32^ By reducing the amount of cholesterol, the mean volume diameter of niosomes decreases (Table 2). This issue has been observed in the study of Mirzaie et al, in preparation of ciprofloxacin niosomes.^34^ Figure 3 displays the vesicle size distribution of all formulations one week after preparation. The formulations containing Span 60/ Tween 60/ cholesterol were chosen for further study and stability evaluation due to their bell-shaped and normal size distribution. Figure 4 illustrates the vesicle size distribution of the formulation over a period of six months. In some formulations, a slight decrease in size was observed over time, which can be attributed to the completion of hydration and the formation of smaller niosomes. Among the formulations, F9 showed the least amount of changes in vesicle size during the six-month storage period, indicating its physical stability. In a study by Sadeghi et al, the formulation containing Span 60/Tween 60/cholesterol (2/1/3 w/w) was found to have the highest physical stability for the preparation of Lysostaphin and LL-37 niosomes.^35^ It has been observed that niosomes containing long alkyl chain (C_18_) surfactants have higher entrapment efficiency and stability compared to those containing shorter chain surfactants (C_12_).^36^ The results related to the investigation of vesicle size distribution and span of selected formulations are presented in Table 2.

**Table 2:** Mean volume diameters (dv50%) and Encapsulation Efficiency percent of SB (measured at 48 h after niosomal preparation) in selected niosomal formulations (Span/Tween 60 containing ones)

**Formulation name**	**dv**_50%_** (µm)±SD**				**Mean span (obtained by Eq. 1)**				**%EE (Mean±SD; n=3)**
	**1 week**	**1 month**	**3 months**	**6 months**	**1 week**	**1 month**	**3 months**	**6 months**	
F 7	10.04 ± 0.03	10.34 ± 0.01	10.57 ± 0.10	11.90 ± 0.38	1.23	1.11	1.33	1.59	59.95 ± 2.02
F 8	7.64 ± 0.04	8.11 ± 0.51	8.07 ± 0.29	8.15 ± 0.30	1.53	6.14	6.42	6.44	65.05 ± 1.36
F 9	6.60 ± 0.12	6.61 ± 0.07	6.54 ± 0.20	6.66 ± 0.07	1.31	1.33	1.05	1.33	66.40 ± 1.11

###  UV analysis

**Figure 5:**
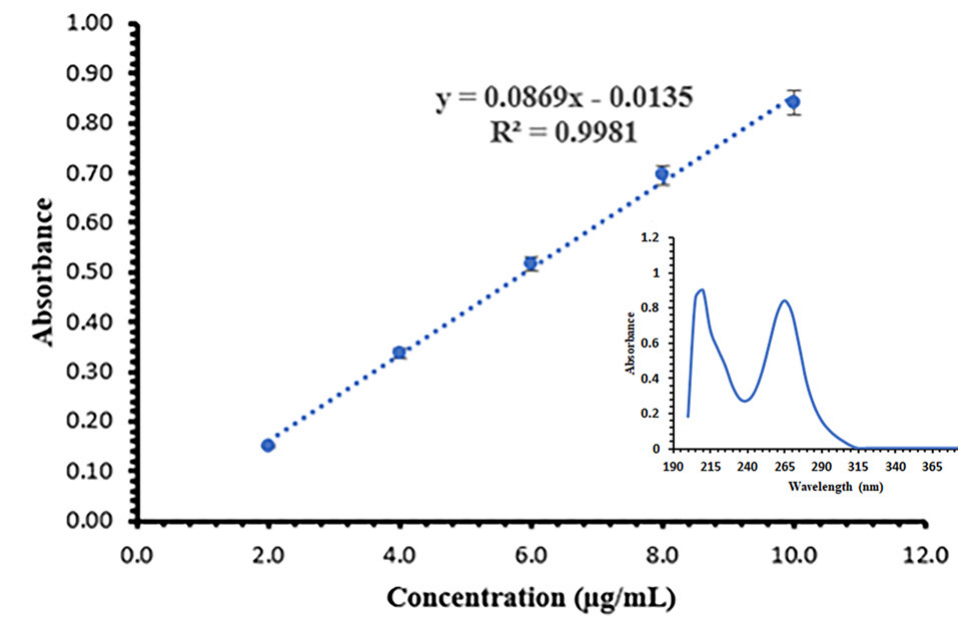
The standard calibration curve and UV absorption spectrum of SB (mean ± SD, n = 6)

 The UV absorption spectrum of the standard solution of SB (10 µg /mL) in the range of 200 to 400 nm is shown in Figure 5. SB had a double peak in the UV absorption spectrum, and the wavelength of 265 nm was chosen to calculate the standard calibration curve because it was in a more favorable range regarding interference with solvents.^19,37^ The standard calibration curve for determining the concentration of SB in the prepared concentrations is shown in Figure 5. The obtained method has appropriate accuracy, and its LOD and LOQ levels were obtained as 0.56 µg /mL and 1.69 µg /mL, respectively. The standard calibration curve equation is given below:


Eq. 6 $$Absorbance=0.0869\;Concentrain\;\left(\mu g/ml\right)-0.0135$$

###  Encapsulation efficiency percent (EE%)

 The results of the EE% evaluation for the selected formulations are presented in Table 2. The lowest and highest EE% were 59.95 ± 2.02 and 66.40 ± 1.11, respectively, in formulations F7 and F9. In a study by Ye et al, they prepared SB nanoliposomes and achieved an EE% of 92%, which is higher than our study. This could be due to the lower amount of drug being encapsulated in the nanoliposome structure. In Ye and colleagues’ study, the concentration of SB in the nano liposomal formulation was 37 μg /mL, while in our study, the SB concentration in niosomal formulations was 100 μg /mL. Additionally, the liposome structures in Ye and colleagues’ study were nano-sized, which could have resulted in the formation of more liposomes and thus a higher EE% in their study.^38^ There was no significant difference in the EE% in formulations F8 and F9. In formulation F8 compared to F7, with the reduction of cholesterol, the percentage of confinement has increased, which can be due to the competition of lipophilic drugs and cholesterol to be placed in the niosome structure.^39-41^ The vesicle size in formulations F8 and F9 were smaller than in formulation F7, but the number of niosomes was more, so there was more space in the bilayer to trap the drug.

###  In vitro release 

**Figure 6:**
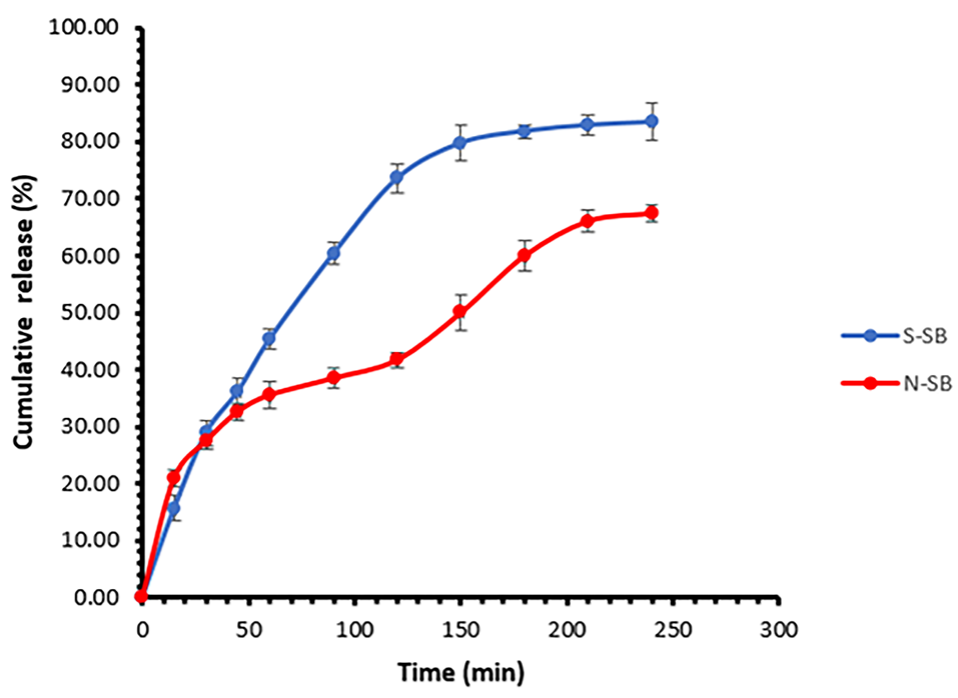
The in vitro release of SB from the Span60/Tween60/cholesterol (45/45/10 mole%) formulation (N-SB) and SB solution (S-SB) (mean ± SD, n = 3)

 The in vitro release of SB from the selected niosomal formulation (F9) was investigated using a Franz diffusion cell and its diagram is presented in Figure 6. The percentage of drug release of niosomal formulation was 61.43 ± 1.42 (mean ± SD, n = 3) after four hours. Additionally, the percentage of soluble drugs passing through the membrane during the same period was 83.43 ± 3.27 (mean ± SD, n = 3). Two phases can be seen in the release diagram of niosomal formulation. At first, the slope of the curve is high and similar to the SB solution, which can be due to the presence of the unentrapped drug. Then the curve slope of the niosomal formulation is lower than that of the SB solution, which can be due to the control of the release rate by the niosome structure and the need for drug diffusion from the noisome structure. In Sadeghi et al study, the prepared lysozyme niosomes had two release phases.^36^ Two phases of drug release from niosomes have also been reported in the study of alpha-lipoic acid niosome preparation by Raeisi Estabragh et al.^17^ The release kinetics in the niosomal formulation follow the Higuchi model (R^2^ = 0.9759, k = 4.357). The Higuchi model describes Fickian diffusion. According to it, the undissolved or encapsulated drug molecules are transformed into the dissolved or free form, causing the boundary to move inward as the dissolved drug molecules diffuse through the outer layer, driven by a concentration gradient.^42,43^ Xiao et al reported the Peppas kinetics for releasing SB from SB and gadolinium co-loaded liposomes.^9^ Based on the Peppas kinetics and n value (0.416), the release mechanism also follows the Fickian diffusion.^44,45^ In the study of Patel et al, SB liposomal dry powder was prepared, and its release was investigated. The release kinetics of the drug from the liposomes followed the Higuchi model.^46^ The results of the release kinetics study are presented in Table 3.

**Table 3:** Parameters values of kinetics models for release of SB from SB niosomes or SB solution

	**SB niosomes (F9)**					**SB solution**				
**Kinetics models**	**R**^2^	**K**	**n**	**Slope**	**Intercept**	**R**^2^	**K**	**n**	**Slope**	**Intercept**
Zero-order	0.9082	0.331		0.2355	15.6180	0.8641	0.451		0.3385	18.359
First order	0.9589	0.005		-0.0018	1.9440	0.9494	0.009		-0.0036	1.946
Higuchi	0.9759	4.357		4.1633	2.2200	0.9666	5.929		6.1040	-1.099
Hixon-Crowell	0.9497	0.007		0.0053	0.2217	0.9284	0.011		0.0092	0.2473
Peppas (Power Law)	0.9645	0.0652	0.416	0.4162	0.8146	0.9656	0.035	0.612	0.6123	0.5383

###  Cytotoxicity assay and flow cytometric study

**Figure 7:**
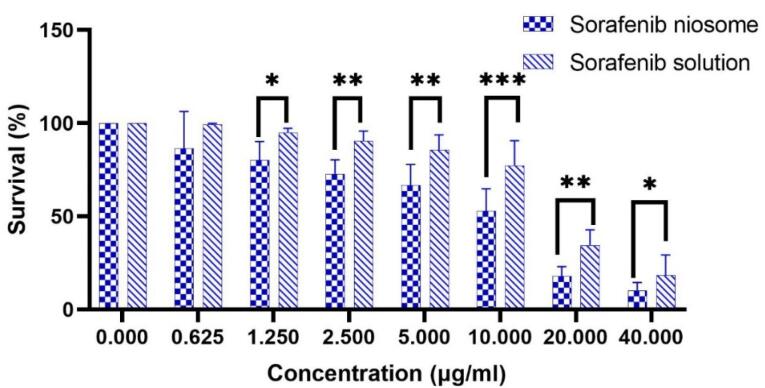
The survival percentage of HepG2 cells in different SB concentrations. * *P* value < 0.05, ** *P* value < 0.001, *** *P* value < 0.0001

**Figure 8:**
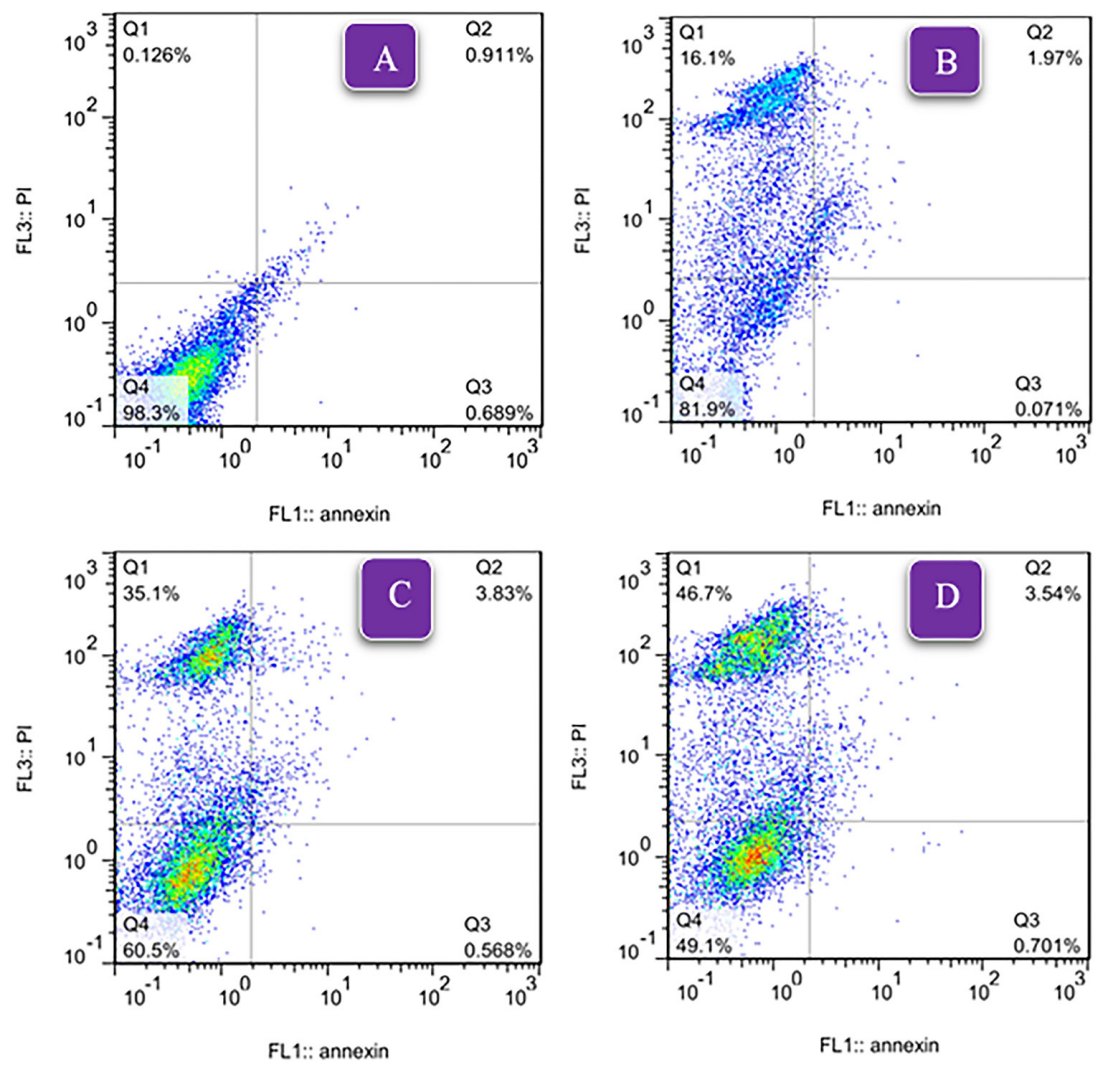
The results of flow cytometric study for HepG2 apoptosis. A: Control group, B: Empty niosome group, C: SB niosome group (7.50 μg /mL), D: SB solution group (16 μg /mL)

 The effect of niosomal formulation (F9) and SB solution on the HepG2 cell line was investigated. Figure 7 shows the survival percentage of cells in different drug concentrations (0-40 µg /mL). The results show a significant difference between the two groups (*P* < 0.05). SB inhibited the growth of the HepG2 cells in a manner that was dependent on the dose. The IC_50_ (95% CI) for niosomal formulation and solution (24 hours after treatment) were obtained at 7.50 µg /mL (5.67 to 9.87) and 15.96 µg /mL (13.78 to 18.53), respectively. It can be concluded that using niosomal formulations effectively lowered the required effective toxic concentration of SB to half. In the study of Wang et al, the IC_50_ for sorafenib in the HepG2 cell line was 17.1 µg /mL.^47^ Also, Cervello et al reported IC_50_ as 19.5 ± 1.4 and 12.0 ± 3.1 µg /mL, 24 and 48 hours after treatment with sorafenib, respectively.^48^ After determining IC_50_, flow cytometry was performed in a concentration equal to IC_50_. The results are shown in Figure 8. Empty niosomes have been used as a blank to eliminate the possible effects of the compounds present in the niosome structure and its production process, and this observed amount of necrosis can be due to the presence of surfactant compounds.^49^ As is evident in the figure, the amount of late apoptosis and necrosis in the SB niosome group (7.50 µg /mL) is slightly higher than in the SB solution group (16 µg /mL). Considering that a lower dose of the drug (approximately half) in niosomal formulation was able to give more apoptosis, it indicates an improvement in the effectiveness of the drug. Yao et al conducted a study on the co-delivery of SB and VEGF-siRNA using pH-sensitive liposomes. After 72 hours, flow cytometry analysis did not reveal a significant difference in the level of apoptosis between the group receiving free SB and the group receiving SB-loaded pH-sensitive carboxymethyl chitosan-modified liposomes. However, when the pH was altered, the amount of apoptosis nearly doubled.^11^

## Conclusion

 Based on the results obtained, it is possible to prepare MLV niosomes of sorafenib with appropriate physical stability and an EE% of approximately 60% using the thin layer film hydration method. The findings from toxicity and flow cytometry studies demonstrate an enhancement in the effectiveness of the niosomal formulation. One of the limitation of this study was the larger size of the niosomes, indicating the need for methods to reduce their size. Future research should be conducted to explore the potential of utilizing a niosomal formulation of SB in clinical trials.

## Competing Interests

 The authors declare that they have no competing financial interests or personal relationships that could have appeared to influence the work reported.

## Ethical Approval

 In this study, the principles of ethics in research have been fully observed and approved by the Research Ethics Committee of Kerman University of Medical Sciences (IR.KMU.REC.1401.057).
